# Widely targeted metabolic analysis revealed the changed pigmentation and bioactive compounds in the ripening *Berchemia floribunda* (Wall.) Brongn. fruit

**DOI:** 10.1002/fsn3.2093

**Published:** 2021-01-20

**Authors:** Liang Shuai, Huan Liu, Lingyan Liao, Tingting Lai, Ziying Lai, Xinxin Du, Zhenhua Duan, Zhenxian Wu, Tao Luo

**Affiliations:** ^1^ College of Food and Biological Engineering/Institute of Food Science and Engineering Technology Hezhou University Hezhou China; ^2^ Sichuan Minzu College Kangding China; ^3^ South China Agricultural University/Guangdong Provincial Key Laboratory of Postharvest Science of Fruits and Vegetables/Engineering Research Center for Postharvest Technology of Horticultural Crops in South China College of Horticulture Ministry of Education Guangzhou China

**Keywords:** anthocyanins, *Berchemia floribunda* (Wall.) Brongn. fruit, flavonoid, phenylpropanoids, widely targeted metabolic analysis; bioactive compounds

## Abstract

*Berchemia* plants were important materials for Chinese traditional medicines due to their special secondary metabolites. Unlike the root, stem and leaf tissues, *Berchemia floribunda* (Wall.) Brongn. fruit was lacked of systematic metabolic investigation. Biochemical analysis found that the total flavonoid and total phenolic content of *Berchemia* fruit pulp showed a peak value at red ripe stage, and then decreased, but the total anthocyanin content sharply increased along with the coloration. By widely targeted metabolomic analysis, 644 metabolites were identified and categorized into 23 groups mainly including flavonoid, organic acids, amino acids, lipids, phenylpropanoid, nucleotides, alkaloids, carbohydrates, alcohols, anthocyanins & proanthocyanidins, vitamins, terpenes, polyphenols, phenolamides, quinones, indole derivatives, and sterides. Among them, 111 metabolites and 123 metabolites respectively showed up‐ and down‐regulation from break stage to full mature. KEGG enrichment analysis indicated that active secondary metabolism such as biosynthesis of phenylpropanoids, flavonoid, and alkaloids happened during *Berchemia* fruit ripening. More importantly, Cyanidin‐3‐*O*‐galactoside and other 3 cyanidins were found to be the predominant pigments in mature *Berchemia* fruit and increased cyanidins and pelargonidins but decreased anthocyanins might be contributed to the purple pigmentation of *Berchemia* fruit. Interestingly, 29 pharmaceutical compounds previously reported in other *Berchemia* tissues were also detected in ripening *Berchemia* fruit pulp: 8 flavonoid, 2 quinones & sucrose showed up‐regulated accumulation while 6 polyphenols, 5 flavonoid, 3 phenylpropanoid, 2 organic acids, 1 quinones and β‐sitosterol showed down‐regulated accumulation In conclusion, our first comprehensive metabolic fingerprint will promote the further study of *B. floribunda* fruit and its medical and food application.

## Graphical abstract

The first comprehensive metabolic fingerprint of B. floribunda fruit was drew by a targeted metabolome. Totally 644 metabolites including 23 groups such as flavonoid, organic acids, amino acids, lipids, phenylpropanoid, nucleotides, and alkaloids, etc, were identified. Active secondary metabolism such as biosynthesis of phenylpropanoids, flavonoid, and alkaloids happened during Berchemia fruit ripening. Cyanidin‐3‐*O*‐galactoside and other 3 cyanidins was the predominant pigments in mature Berchemia fruit and increased cyanidins and pelargonidins but decreased anthocyanin might result in purple pigmentation of Berchemia fruit.

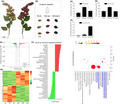

## 1 INTRODUCTION

The root, stem, vine, leaf and whole plant of some *Berchemia* (Rhamnaceae) species have been used in Chinese traditional medicines (In Directory of Chinese Materia Medica, 1986; Inoshiri et al., 1987; Kang et al., 2017). These *Berchemia* plants were reported to relieve pain, act as expectorant, antipyretic and be used for treatment of gall stones, liver disease, rheumatic arthritis, tuberculosis (TB), acute or chronic tracheitis, jaundice, diarrhea and carbuncle (In Directory of Chinese Materia Medica, 1986; Inoshiri et al., 1987; Kang et al., 2017). The *Berchemia* (Rhamnaceae) comprises 32 deciduous plants worldwide which were mainly located in temperate and tropical areas in Asia.**^1^** Among them, 18 species and 6 varieties were distributed in south, southwest, central south and east of China (Chen & Dong, 2006). The dried root of *Berchemia lineata* (L.) DC). was named as Tiebaojin, Huangshanteng, Goujiaoli, Tiyuncao or Laoshucao in traditional Chinese medicine (Wei et al., 2015). Previous researches indicated that the stem, vine and root materials used for Chinese traditional medicine “Tiebaojin” were actually from more than 4 *Berchemia* species including *Berchemia lineata* (L.) DC.), *Berchemia polyphylla Wall. ex Laws*, *Berchemia polyphylla* var. *leioclada* Hand. ‐Mazz) and *Berchemia floribunda (Wall.) Brongn* (Teng et al., 2010). Although the tissues used for medicine were produced from different plants of *Berchemia* genera and their medical chemical constituents might be distinct, the dominant metabolites in these materials were commonly flavonoids and flavonoid glycosides, phenols and phenolic glycosides (Shen et al., 2010), lignans, quinones and their dimer forms, and terpenes (Wei et al., 2015). At present, many pharmaceutical compounds had been separated from stem, leaves, wood, root, barks and whole plant of these *Berchemia* genera, but little is known about the chemical constituents of the *Berchemia* fruits which was used in food coloring and Tibetan medicine (Kang et al., 2017).

The largest group of secondary metabolites found in *Berchemia* plants was flavonoid. The flavonols such as quercetin, dihydroquercetin, quercetin 3‐α‐arabinofuranoside, rutin (quercetin 3‐*O*‐rutinoside), kaempferol, aromadendrin (dihydrokaempferol), kaempferol 3‐*O*‐glucoside and myricetin 3‐*O*‐rhamnoside, a flavone (4, 2′, 4′, 6′‐Tetrahydroxychalcone), two flavanones (eriodictyol, naringenin), and two flavonoids (5, 7‐dihydroxychromone and narcissoside) were isolated and identified from *Berchemia racemosa* SIEB ZUCC, *Berchemia formosana* Schneider, *Berchemia zeyheri* Sond., *Berchemia polyphylla* var. *Leioclada*, *Berchemia floribunda* (Wall.) Brongn. And *Berchemia lineata* (L.) DC.). In addition, polyphenols such as gallocatechin, catechin, epigallocatechin, epicatechin, protocatechuic acid and protocatechuic acid *O*‐glucoside were found to be abundant in these *Berchemia* plants. Phenylpropanoid such as ferulic acid, vanillic acid, phillygenin, quinones such as emodin, chrysophanic acid, and aurantio‐obtusi, organic acids (4‐Hydroxybenzoic acid, syringic acid *O*‐glucoside), and β‐sitosterol were also isolated from these *Berchemia* plants. Although more than 30 metabolites were isolated and investigated in the root, stem, vine, leaf and whole plant of *Berchemia* plants, limited information is reported about the chemical constituents of *Berchemia* fruits.

In recent years, liquid chromatography–mass spectrometry (LC–MS)‐based metabolomics has been facilitated by the construction of MS2 spectral tag (MS2T) library from the total scan ESI MS/MS data, and the development of widely targeted metabolomic method using MS/MS data gathered from authentic standards (Chen et al., 2013). In recent years, UPLC‐ESI‐MS/MS based widely targeted metabolomic method has been widely applied in plant metabolite analysis in maize (Wen et al., 2014), rice (Chen et al., 2014; Chen et al., 2013; Dong et al., 2014), tomato (Zhu et al., 2018), sweet potato (Wang, Li, et al., 2018`), fig (Wang, Cui, et al., 2017), sesame (Wang, Zhang, et al., 2018), strawberry (*Fragaria* × *ananassa*) (Paolo et al., 2018), asparaguses (Dong et al., 2019), citrus (Wang et al., 2016, 2019; Wang, Yang, et al., 2017), potato (Cho et al., 2016), buckwheat (Li et al., 2020), tea (Zheng et al., 2019; Zhu et al., 2020; Wu et al., 2020), wheat (Chen et al., 2020), pepper and other plants (Ginkgo, Meng et al., 2019; *Phalaenopsis amabilis*, Meng et al., 2020; Qingke, Zeng et al., 2020). In the place of origin, the *Berchemia* fruits were usually not harvested according to their grade of maturity. The differences in metabolic components of fruits with different ripenesses had not caught enough attention and not been compared. In this study, we analyzed the secondary metabolites of *Berchemia floribunda (Wall.) Brongn*. fruits from break stage (start coloring) and full‐mature stage by a widely targeted metabolomic method using HPLC‐ESI‐triple quadrupole‐linear ion trap. We further screened out and annotated the significantly differently accumulated metabolites (DAM) in *Berchemia* fruits during the ripening process. Our comprehensive metabolic fingerprint was expected to guide the maturity grading of *Berchemia floribunda* (Wall.) Brongn. fruits and their further applications in food and pharmaceutical industry.

## 2 MATERIALS AND METHODS

### 2.1 Fruit materials

The Gou‐er‐cha (*Berchemia floribunda* (Wall.) Brongn.) fruits were harvested from the mountainside at an altitude of 2000 m located in Danba town, Ganzi Tibetan Autonomous Prefecture, Sichuan Province, China. The harvested fruits were immediately taken to the laboratory and graded according to maturity and coloring stages: break (B), red ripe (RP), and full‐mature (FM) stage. After the removal of seeds, the pulp of fruit was immediately frozen in liquid nitrogen and stored at −80°C until be used.

### 2.2 Chemicals

Acetic acid, methanol, and acetonitrile were HPLC degrade (Merck & Co., Inc.). Ultrapure water was prepared by distilled water through a Milli‐Q A10 system (Millipore). Ethanol, Folin‐Ciocalteau reagent, Na_2_CO_3_, gallic acid, sodium nitrite, aluminum nitrate, sodium hydroxide, rutin, and gallic acid were all analytical reagents and supplied by Sinopharm Chemical Reagent Co., Ltd.

### 2.3 Determinations of total phenolics, flavonoid, and anthocyanin contents

The ethanolic extract used for determination of total phenolics and flavonoid contents were prepared as follows: the frozen sample was ground into powder in liquid nitrogen; 0.1 g powder was added into 3 ml 80% ethanol in 10 ml tuber and then extracted under a ultrasonication for 30 min (with a ice bath to cool); after a centrifugation at 5,000 *g* for 5 min, the supernatant was transfered into a 10 ml volumetric flask. The residue was then extracted twice with 3 ml 80% ethanol as described above. The combined ethanolic extract in volumetric flask was adjusted to 10 ml using 80% ethanol. The ethanolic extract was stored at amber colored air‐tight containers at 4°C.

The total phenolic content (TPC) was determined by the Folin–Ciocalteu method (Pastrana‐Bonilla et al., 2003). 0.25 ml ethanolic extract (or standard solution of gallic acid) was added into 5.75 ml deionized water in a 25 ml amber volumetric flask, then mixed with 0.5 ml Folin–Ciocalteau reagent. 2 min later, 1.5 ml 20% Na_2_CO_3_ was added and fully mixed. The solution was adjusted to 25 ml using 80% ethanol and kept under dark for 30 min. The optical density of the blue‐colored samples was measured at 760 nm. The total phenolic contents were calculated according to the standard curve and expressed as mg gallic acid equivalent (GAE)/g fresh weight. The assay was subjected to three repeats.

The total flavonoid contents were measured using a modified colorimetric method (Jia et al., 1999; Liu et al., 2008). The ethanolic extract solution was diluted by three folds. Then, 1 ml diluted ethanolic extract was added to a test tube containing 4 ml of 80% ethanol. Sodium nitrite solution (5%, 0.5 ml) was added to the mixture and maintained for 6 min. Then, 0.5 ml of 10% aluminum nitrate was added, fully mixed and maintained for 6 min. 0.5 ml of 1 M sodium hydroxide was finally added and fully mixed maintained for 10 min. The absorbance of the mixture at 510 nm was measured immediately in comparison to a standard curve prepared by rutin. The flavonoid contents were expressed as mg rutin equivalent (RE)/g fresh weight.

The anthocyanin contents were measured and calculated according to a colorimetric method (Fuleki & Francis, 1968).

$$C\left(\text{mg}/g\right)=\frac{A_{536\text{nm}}\times V\times N}{98.2\times m}$$

Note: *A*_536nm_: absorbance at 536 nm; *V*: constant volume before test, *N*: dilution times, extinction coefficient for anthocyanin was 98.2, *m*: sample mass.

### 2.4 Widely targeted metabolomic analysis

#### 2.4.1 Sample extraction

The frozen pulp was crushed using a mixer mill (MM 400; Retsch, Germany) with a zirconia bead for 1.5 min at 30 Hz. Sample powder of 100 mg was weighted and extracted overnight at 4°C with 1.0 ml 70% aqueous methanol, vortexed for three times during the period to increase the extraction efficiency. After be centrifuged at 10,000 *g* for 10 min, the supernatant was collected, passed through a Carbon‐GCB SPE Cartridge (250 mg, 3 ml, CNWBOND, ANPEL). Before LC‐MS analysis, each sample was filtrated (SCAA‐104, 0.22 μm pore size; ANPEL, http://www.anpel.com.cn/).

#### 2.4.2 UPLC Separation

After the filtering, 2 μl sample was injected and analyzed using an ultraperformance liquid chromatography (Shim‐pack UFLC CBM30A system, SHIMADZU, Japan) coupled with tandem ESI‐MS/MS (6500 Q‐TRAP, Applied Biosystems). The UPLC conditions were performed according to a previous reported method (Wang, Li, et al., 2018; Wang, Zhang, et al., 2018). The metabolites were separated by an ACQUITY UPLC HSS T3 column (C_18_, 100 mm × 2.1 mm i.d., 1.8 µm, Waters). Mobile phase was composed of phase A (ultrapure water containing 0.04% acetic acid) and phase B (acetonitrile containing 0.04% acetic acid). The elution program was performed as follows (min, % A): (0, 95), (11.0, 5), (12, 5), (12.1, 95), (15, 95). The flow rate was 0.40 ml/min, and the column temperature was kept at 40°C. The effluent was alternatively connected to the ESI‐triple quadrupole‐linear ion trap (Q‐TRAP)‐MS.

#### 2.4.3 ESI‐Q TRAP‐MS/MS

The Mass spectrometry was according to the previous reported method for analyzing widely targeted metabolites (Chen et al., 2013). LIT and triple quadrupole (QQQ) scans were acquired using a triple quadrupole‐linear ion trap mass spectrometer (Applied Biosystems 6500 QTRAP). The MS/MS system was equipped with an ESI Turbo Ion Spray interface, operating in a positive ion mode and controlled by Analyst 1.6.3 software (AB Sciex, Waltham, MA, USA). The ESI source operation parameters were as follows: ion source, turbo spray; source temperature 500°C; ion spray voltage (IS) 5,500 V; ion source gas I (GSI), gas II (GSII), curtain gas (CUR) were set at 55, 60, and 25.0 psi, respectively; the collision gas (CAD) was high. Instrument tuning and mass calibration were performed with 10 and 100 μM polypropylene glycol solutions in QQQ and LIT modes, respectively. QQQ scans were acquired as MRM experiments with collision gas (nitrogen) set to 5 psi. DP and CE for individual MRM transitions was done with further DP and CE optimization. A specific set of MRM transitions were monitored for each period according to the metabolites eluted within this period.

#### 2.4.4 Qualitative and quantitative analysis of metabolites

After removal of the isotope signal and the repetitive signal, metabolites were qualitative by the secondary spectral information based on the public metabolite database (e.g., MassBank, KNApSAcK…) and the self‐built database MetWare database (from Metware Biotechnology Co., Ltd.).

The metabolites were quantified using multiple reaction monitoring (MRM) of triple quadrupole mass spectrometry. The ions corresponding to other molecular weight substances were excluded, and the precursor ions of the target substance were screened. Meanwhile, in the collision cell, the precursor ions were ionized to break and form fragment ions, and the characteristic fragment ions were selected by triple quadrupole filtration. This makes the quantitative results more accurate and repeatable (Fraga et al., 2010). The mass spectrometry files were opened with MultiaQuant software 3.0.3 to carry out the integration and correction of chromatographic peaks, and the relative content of the corresponding substance in the peak area of each chromatographic peak was calculated (Wang et al., 2019).

#### 2.4.5 PLS‐DA and screening of differential accumulated metabolites (DAM)

The metabolites which were not detected in more than two repeats at any stage (B or FM stage) were filtered out. The rest metabolites were used for Orthogonal Partial Least Squares‐Discriminant Analysis (OPLS‐DA) (Eriksson et al., 2006). The metabolites with VIP value ≧1, |log_2_(FM/B)|≧1 and *p*‐value < .05 (*t* tests) were screen out as DAMs. The annotation of all of the metabolites by KEGG database (Kanehisa & Goto, 2000) were manual examined. The enrichment analysis of DAMs, up‐regulated and down‐regulated were conducted by the perform Metabolites Biological Role (MBROLE) 2.0 (López‐Ibáñez et al., 2016).

### 2.5 Statistical analysis

The variance of data was analyzed using SPSS software package release 18.0 (SPSS Inc.). Multiple comparisons were performed by One‐way ANOVA based on Duncan's multiple range tests, while paired‐samples *t* tests were performed to test the statistical significance between two samples.

## 3 RESULTS AND DISCUSSION

### 3.1 Determination of total flavonoid, total phenolic and total anthocyanin contents

**FIGURE 1:**
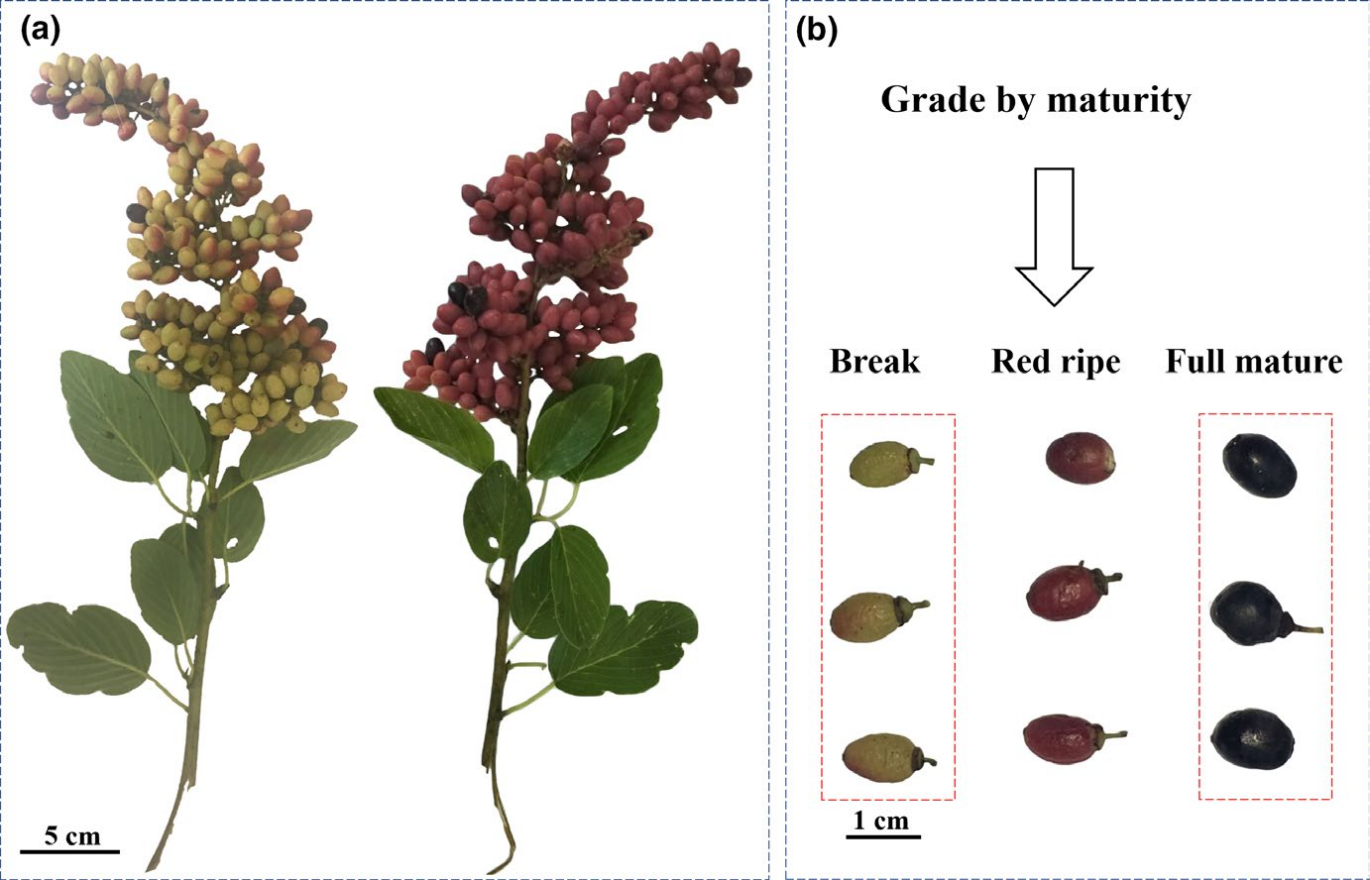
*Berchemia floribunda* fruits with different maturity. Red frame: the fruits from break and full‐mature stage were used for LC‐MS/MS analysis of metabolites

**FIGURE 2:**
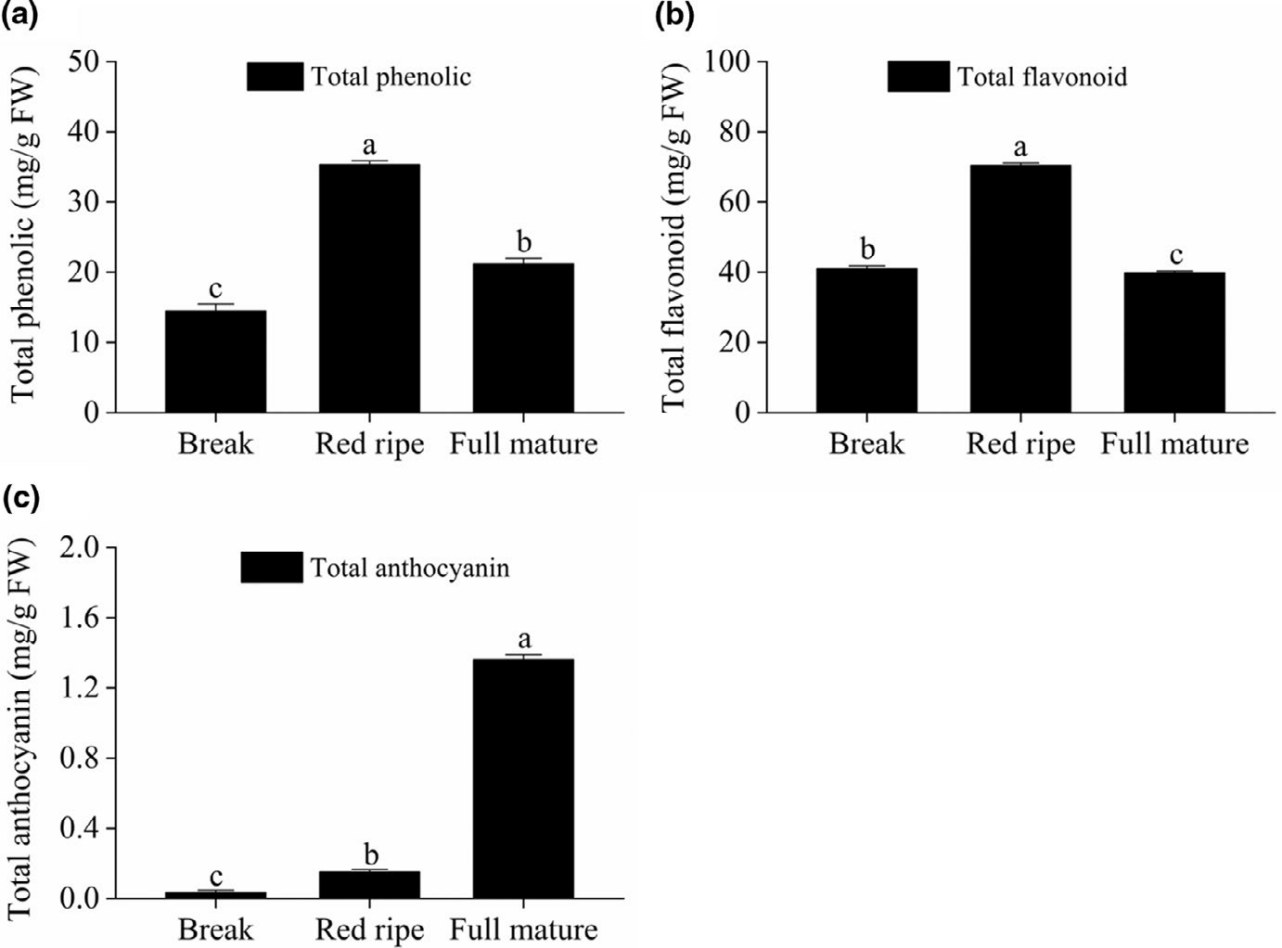
The total phenolic, total flavonoid and total anthocyanin content in pulp of *Berchemia floribunda* fruit at break, red ripe, and full‐mature stage

The *Berchemia* fruits showed a yellowish‐pink pigmentation at break stage and turned to be red at red ripe stage. It was very interesting to note that the fruit color further turned to be purple black at the full‐mature stage (Figure 1). In order to investigate the level of bioactive compounds, we determined the total phenolic, total flavonoid, and total anthocyanin content in pulp of *Berchemia* fruits at break, red ripe and full‐mature stage. As shown in Figure 2a, the total phenolic content of *Berchemia* fruit pulp at break, red ripe, and full mature was respectively 14.51, 35.32 and 21.18 mg/g FW. Similarly, the total flavonoid content was 40.93 mg/g FW at break stage, increased to 70.44 mg/g FW at red ripe stage but then fell back to 39.88 mg/g FW at full‐mature stage (Figure 2b). The total anthocyanin content was 0.035 mg/g FW at break stage, then continuous increased along with the coloration of the *Berchemia* fruit and rose to 1.36 mg/g FW at full‐mature stage (Figure 2c). Thus, the total anthocyanins accumulated along with maturity and showed different change trend compared to flavonoids and phenolics.

### 3.2 Identification, quantification and classification of metabolites detected in break and full‐mature *Berchemia* fruits

**FIGURE 3:**
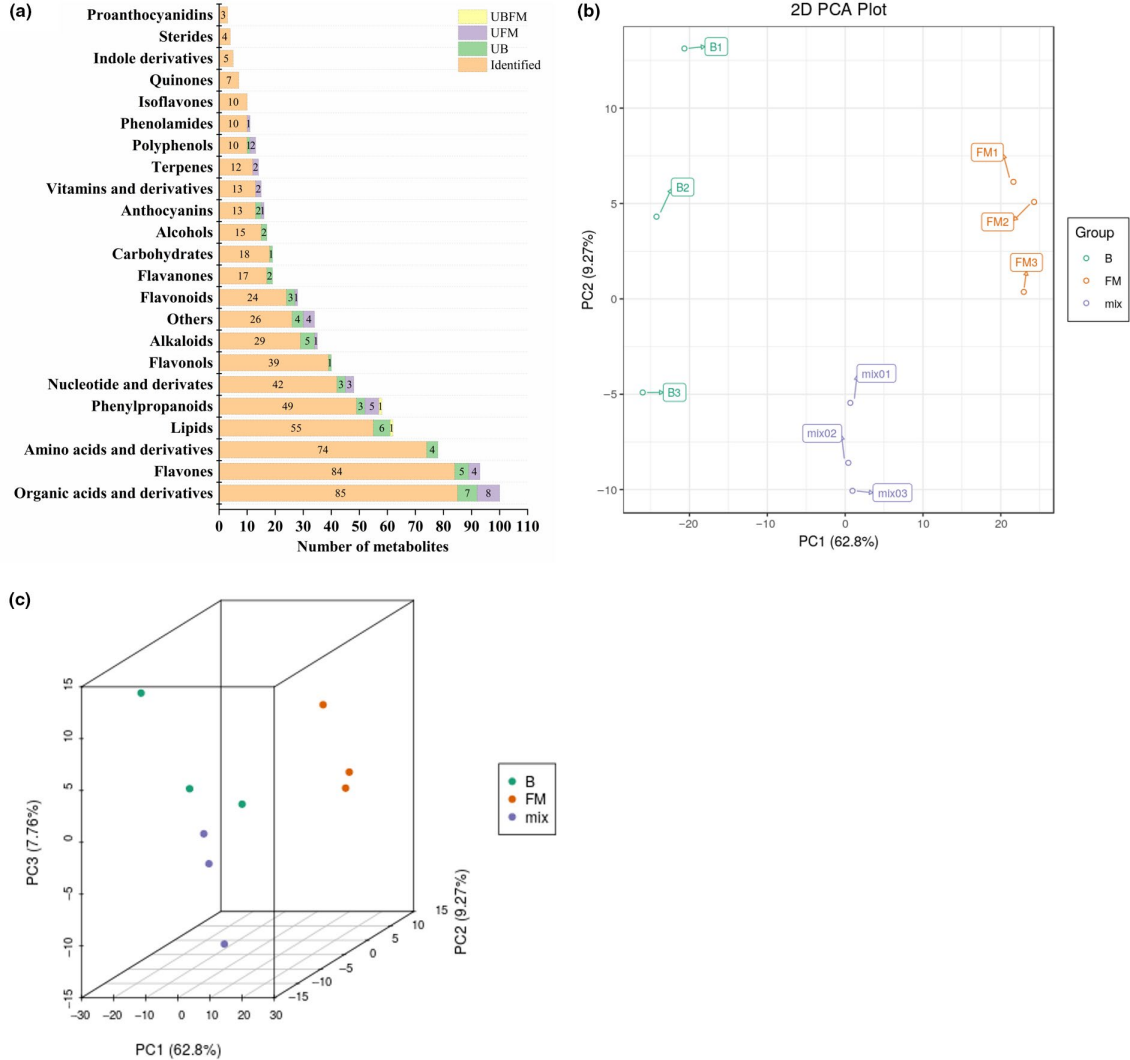
Category of 730 detected metabolites (a) and the PCA analysis of *Berchemia floribunda* fruit samples (b, 2D PCA; c, 3D PCA) at B and FM stages. BFM: metabolites detected in both of the break (B) and full‐mature (FM) fruits; UB: metabolites undetected in two or three repeats of the break (B) fruit samples; UFM: metabolites undetected in two or three repeats of the full‐mature (FM) fruit samples; UBFM: metabolites undetected in two or three repeats of the break fruit samples and undetected in two or three repeats of the full‐mature fruit samples; mix: the quality control samples

In order to compare the metabolic finger‐print of fruits at break stage to that of fruits at full‐mature stage, a HPLC‐ESI‐triple quadrupole‐linear ion trap (Q‐TRAP)‐MS analysis was used to identify and quantify the metabolites in *Berchemia* fruit pulp. In total, 730 metabolites were detected in *Berchemia* fruit pulp. It was worthy to note that 49 metabolites containing 7 organic acids and derivatives, 6 lipids, 5 alkaloids, 5 flavones, 4 phenylpropanoids, 4 amino acids and derivatives, 4 others, 3 nucleotide and derivatives, 2 flavonoids, 2 flavanones, 2 alcohols, 2 anthocyanins, 1 flavonol, 1 carbohydrate and 1 polyphenols were undetected in two or three repeats of the break fruit pulp (named as UB); while 36 metabolites containing 8 organic acids and derivatives, 5 phenylpropanoids, 4 flavones, 4 others, 3 nucleotide and derivatives, 2 phenolamides, 2 polyphenols, 2 terpenes, 2 vitamins and derivatives, 1 isoflavone, 1 anthocyanin, 1 flavonoid and 1 alkaloid were undetected in two or three repeats of the full‐mature (FM) fruit pulp (named as UFM); One metabolite (DGMG (18:2) isomer 2) was undetected both in two repeats of the break fruit samples and two repeats of the full‐mature fruit samples (named as UBFM) (Figure 3a). Thus, the above mentioned 86 metabolites containing UB, UFM, and UBFM were excluded and the rest 644 metabolites categorized into 23 groups were used for further analysis.

The largest group of metabolites identified in the pulp of *Berchemia* fruit was flavonoid which containing 95 flavones, 40 flavanols, 27 flavonoids, 20 flavanones, and 11 isoflavone. Moreover, 100 organic acids and derivatives, 78 amino acids and derivatives, 63 lipids, 58 phenylpropanoids, 49 nucleotide and derivates, 36 alkaloids, 19 carbohydrates, 18 alcohols, 16 anthocyanins, vitamins and derivatives, 14 terpenes, 13 polyphenols, 12 phenolamides, 7 quinones, 5 indole derivates, 5 sterides, and 3 proanthocyanidins were detected in *Berchemia* fruit pulp (Figure 3a). In further, 34 others metabolites such as d‐glucoronic acid, gluconic acid, d‐glucose‐6‐phosphate disodium salt, hinokitiol, mangiferin were detected in *Berchemia* fruit pulp (Figure 3a). As shown in Figure 3a,b, 2D and 3D PCA (principal Component Analysis) demonstrated the significant and authentic differences among the samples at break stage, samples at full‐mature and quality controls (QC, mixed samples).

### 3.3 Significantly differently accumulated metabolites (DAMs) during the ripening process of *Berchemia* fruits

**FIGURE 4:**
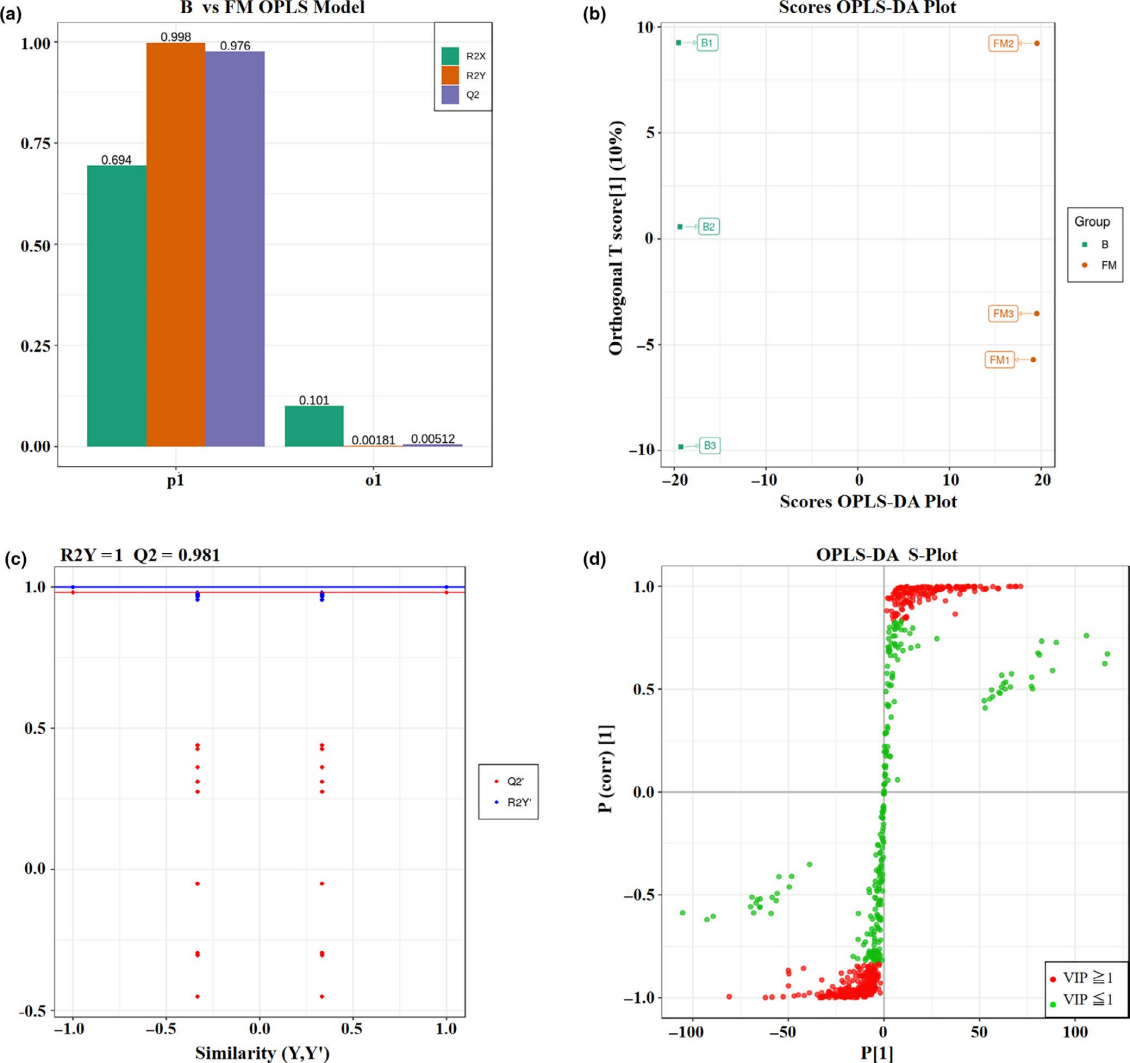
OPLS‐DA model (a), score OPLS‐DA plot (b), validation of OPLS‐DA model (c), OPLS‐DA S‐Plot (d)

**FIGURE 5:**
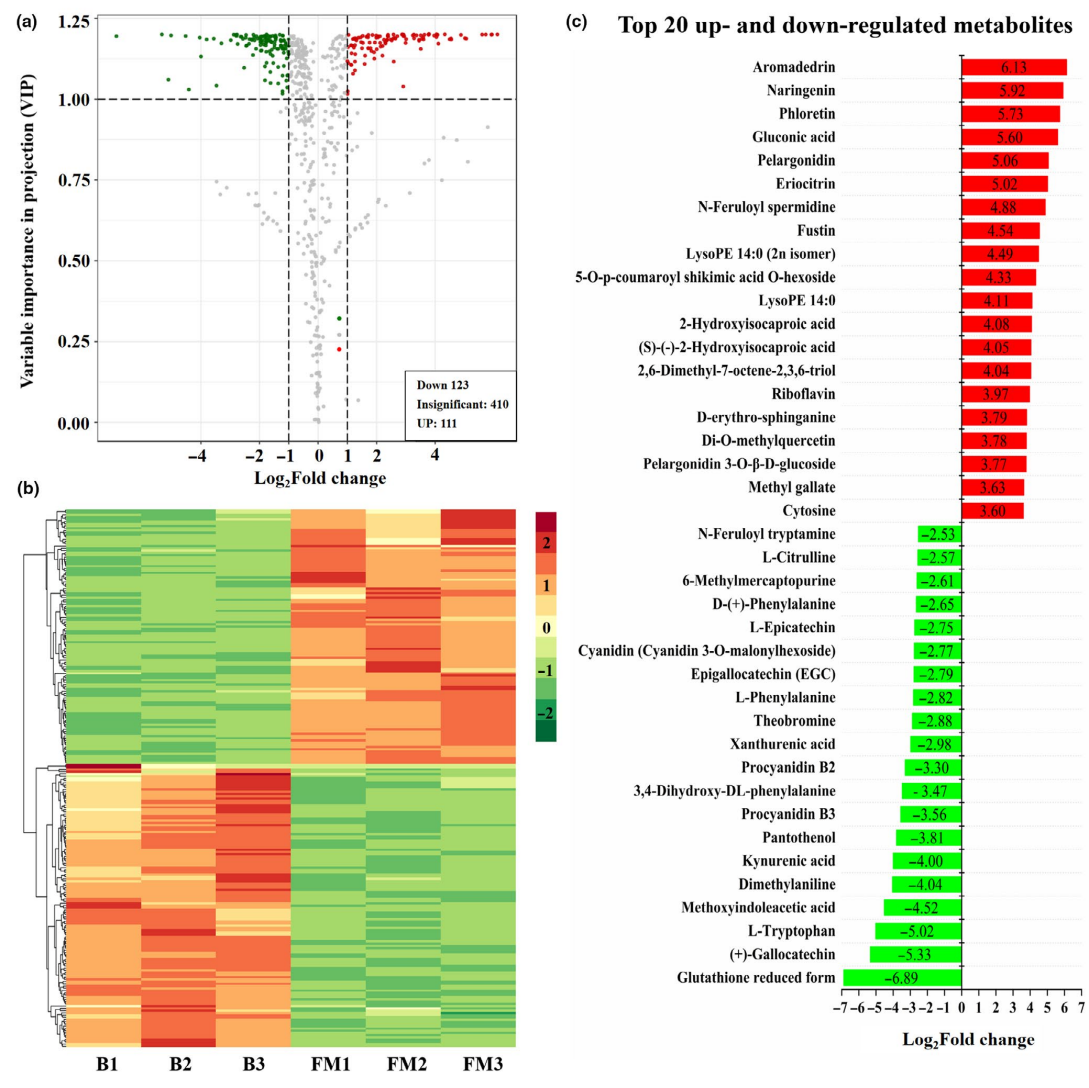
Volcano map (a), cluster heat map analysis (b) and the top 20 (c) of DAMs (FM vs. B) (c). DAMs: red points represent DAMs with VIP≧ 1 and log_2_(FM vs. B)≧1 (*p*‐value <0.05), green points represent DAMs with VIP≧ 1 and log_2_(FM vs. B)≦–1 (*p*‐value <0.05); gray points represent insignificantly changed metabolites

Based on the results of Orthogonal Projections to Latent Structures‐Discriminant Analysis (OPLS‐DA) and significance difference analysis, the significantly differently accumulated metabolites (DAMs) were screened. The metabolites with variable importance in projection (VIP) value ≥ 1, fold change (FM vs. B) ≥ 2 or fold change (FM vs. B) ≤ 0.5, and *p*‐value (*t* test FM vs. B) < 0.05 were identified as the DAMs in *Berchemia* fruits from break to full‐mature stage (Figure 4a–d). In details, 123 metabolites and 111 metabolites were respectively identified as down‐regulated and up‐regulated DAM during the ripening process (Figure 4d; Figure 5a). The expression of DAMs showed significant clustering groups: some DAMs were significantly up‐regulated but the others down‐regulated in fruits at FM stages (Figure 5b).

The top 20 significantly down‐regulated metabolites contained 6 amino acid and derivatives, 3 polyphenols, 2 organic acids and derivatives, 2 alkaloids, 2 proanthocyanidins, 1 alcohol, 1 anthocyanin, 1 nucleotide and derivates, 1 indole derivatives, and 1 others; these DAMs were respectively reduced glutathione, (+)‐gallocatechin (GC), l‐tryptophan, methoxyindoleacetic acid, dimethylaniline, kynurenic acid, pantothenol, procyanidin B3, 3,4‐dihydroxy‐DL‐phenylalanine, procyanidin B2, xanthurenic acid, theobromine, l‐phenylalanine, epigallocatechin (EGC), cyanidin 3‐*O*‐malonylhexoside, l‐epicatechin, d‐(+)‐phenylalanine, 6‐methylmercaptopurine, l‐citrulline, and N‐feruloyl tryptamine.

The top 20 significantly up‐regulated metabolites contained 3 flavonols, 3 flavanones, 3 organic acid derivatives, 2 anthocyanins, 2 lipids, 1 amino acid, 1 flavonoid, 1 vitamin, 1 alcohol, 1 flavone, 1 phenolamides, and 1 Others. The highest up‐regulated metabolite was aromadedrin (dihydrokaempferol). The rest top up‐regulated DAMs were naringenin, phloretin, Gluconic acid, Pelargonidin, Eriocitrin, N‐Feruloyl spermidine, Fustin, LysoPE 14:0 (2n isomer), 5‐*O*‐p‐coumaroyl shikimic acid *O*‐hexoside, LysoPE 14:0, 2‐Hydroxyisocaproic acid, (S)‐(‐)‐2‐hydroxyisocaproic acid, 2,6‐dimethyl‐7‐octene‐2,3,6‐triol, riboflavin, d‐erythro‐sphinganine, Di‐O‐methylquercetin, pelargonidin 3‐*O*‐beta‐d‐glucoside (Callistephin chloride).

### 3.4 The enriched pathways of up‐regulated and down‐regulated DAMs

The number and percentage of up‐regulated and down‐regulated DAMs in identified compounds were analyzed (Table 1). More than 30% of the identified alcohols, amino acid & derivatives, anthocyanins, carbohydrates, flavanone, flavonoid, lipids, indole derivatives, organic acids & derivatives, phenolamides, phenylpropanoids, polyphenols, proanthocyanidins, and vitamins & derivatives showed differently accumulation from B to FM stage. Its was worthy to note that most of the DAMs of alkaloids, amino acid & derivatives, flavone, and phenylpropanoids, and all the DAMs of indole derivatives, polyphenols, proanthocyanidins and quinones showed a up‐regulation during the maturation process of *Berchemia* fruit. On the other hand, most of the DAMs of anthocyanins and all the DAMs of carbohydrates, isoflavone and lipids showed a down‐regulation from B to FM stage.

**TABLE 1:** The number and percentage of up‐regulated and down‐regulated DAMs in identified compounds

Class	Up‐regulated DAMs (%)	Down‐regulated DAMs (%)
Alcohols	3 (20.0%)	4 (26.7%)
Alkaloids	7 (24.1%)	1 (3.4%)
Amino acid & derivatives	21 (28.4%)	6 (8.1%)
Anthocyanins	1 (7.7%)	3 (23.1%)
Carbohydrates	−	10 (55.6%)
Flavanone	3 (17.7%)	4 (23.5%)
Flavone	17 (20.2%)	7 (8.3%)
Flavonoid	5 (20.8%)	4 (16.7%)
Flavonol	5 (12.8%)	5 (12.8%)
Isoflavone	−	1 (10.0%)
Lipids	−	27 (49.1%)
Indole derivatives	3 (60.0%)	−
Nucleotide and derivates	5 (11.9%)	7 (16.7%)
Organic acids & derivatives	20 (23.5%)	19 (22.4%)
Others	6 (23.1%)	3 (11.5%)
Phenolamides	2 (20.0%)	1 (10.0%)
Phenylpropanoids	12 (24.5%)	4 (8.2%)
Polyphenol	5 (50.0%)	−
Proanthocyanidins	2 (66.7%)	−
Quinones	1 (14.3%)	−
Terpene	1 (8.3%)	2 (16.7%)
Vitamins & derivatives	4 (30.8%)	3 (23.1%)

**FIGURE 6:**
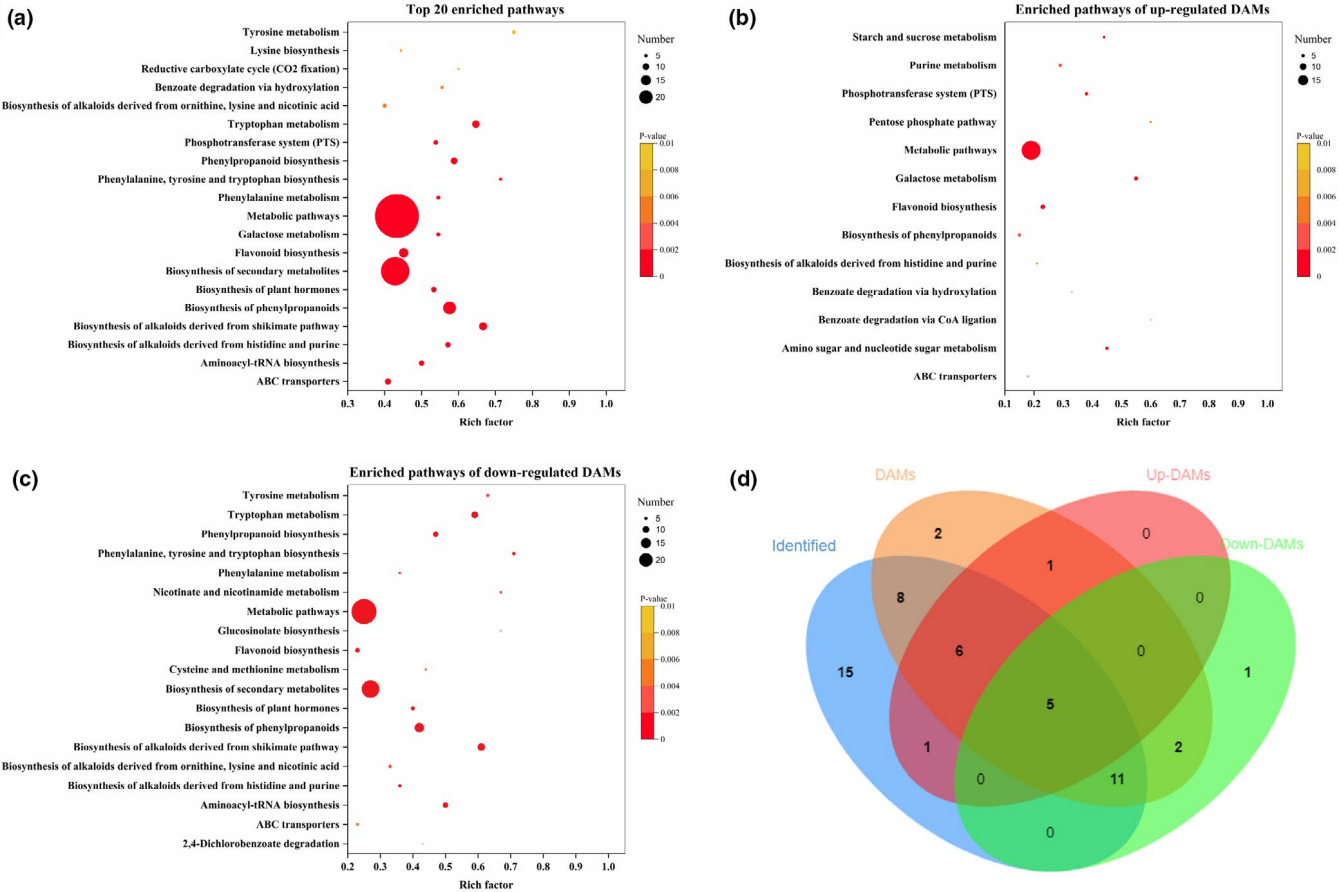
KEGG enrichment analysis and venn chart of enriched pathways. (a) the top 20 enriched pathways of all DAMs; (b) enriched pathways of up‐regulated DAMs; (c) enriched pathways of down‐regulated DAMs; (d) venn picture showing enriched pathways of the identified compounds, all DAMs, up‐regulated DAMs and down‐regulated DAMs

Among the 644 identified compounds, 454 compounds (containing 16 compounds with repeated annotation) were able to be annotated by KEGG compound IDs. Moreover, 76 compounds from the 111 up‐regulated DAMs and 85 compounds from the 123 down‐regulated DAMs were respectively annotated by KEGG compound IDs. The DAMs were significantly enriched (*p*‐value < .05) into 34 pathways and the top 20 pathways including ABC transporters, aminoacyl‐tRNA biosynthesis, biosynthesis of alkaloids derived from histidine and purine, biosynthesis of alkaloids derived from shikimate pathway, biosynthesis of phenylpropanoids, biosynthesis of plant hormones, biosynthesis of secondary metabolites, flavonoid biosynthesis, galactose metabolism, metabolic pathways, phenylalanine metabolism, phenylalanine, tyrosine and tryptophan biosynthesis, phenylpropanoid biosynthesis, phosphotransferase system (PTS), tryptophan metabolism, biosynthesis of alkaloids derived from ornithine, lysine and nicotinic acid, benzoate degradation via hydroxylation, reductive carboxylate cycle (CO_2_ fixation), lysine biosynthesis and tyrosine metabolism (Figure 6a).

The up‐regulated DAMs were significantly enriched (*p*‐value < 0.05) into 14 pathways including flavonoid biosynthesis, galactose metabolism, metabolic pathways, phosphotransferase system (PTS), starch and sucrose metabolism, amino sugar and nucleotide sugar metabolism, purine metabolism, biosynthesis of phenylpropanoids, pentose phosphate pathway, biosynthesis of alkaloids derived from histidine and purine, benzoate degradation via hydroxylation, ABC transporters, and benzoate degradation via CoA ligation (Figure 6b).

The down‐regulated DAMs were significantly enriched (*p*‐value < .05) into 19 pathways including biosynthesis of phenylpropanoids, tryptophan metabolism, phenylpropanoid biosynthesis, biosynthesis of alkaloids derived from shikimate pathway, aminoacyl‐tRNA biosynthesis, metabolic pathways, phenylalanine, tyrosine and tryptophan biosynthesis, flavonoid biosynthesis, biosynthesis of alkaloids derived from histidine and purine, biosynthesis of plant hormones, biosynthesis of secondary metabolites, biosynthesis of alkaloids derived from ornithine, lysine and nicotinic acid, nicotinate and nicotinamide metabolism, phenylalanine metabolism, tyrosine metabolism, cysteine and methionine metabolism, ABC transporters, 2,4‐Dichlorobenzoate degradation, and glucosinolate biosynthesis (Figure 6c).

The venn diagram showed that 5 pathways including ABC transporters, biosynthesis of alkaloids derived from histidine and purine, biosynthesis of phenylpropanoids, flavonoid biosynthesis and metabolic pathways were the common enriched pathways among the identified compounds, DAMs, up‐regulated DAMs and down‐regulated DAMs (Figure 6d). the above results indicated active secondary metabolism including pathways related to phenylpropanoids, flavonoid and alkaloids during the ripening of *Berchemia* fruit.

### 3.5 The changes of pigment compounds related to coloration of *Berchemia* fruit

Few large‐scale investigation of the pigments in the *Berchemia* fruit was reported. According to a previous results of TLC, the main pigments in *Berchemia* fruit were deduced to be pelargonidin 5‐glucoside and pelargonidin 3‐glucoside‐5‐rutinoside (Zhou, 2000). The yellowish‐pink *Berchemia* fruits was turned to be red at red ripe stage and then be purple at full mature. In this experiment, sixteen anthocyanins (including 5 cyanidins, 4 peonidins, 3 pelargonidin, 1 petunidin, 1 rosinidin and 1 delphinidin) and 3 proanthocyanidins (including procyanidin A2, procyanidin B2 and procyanidin B3) were detected in *Berchemia* fruits (Table 2). The anthocyanin with highest abundance (10^8^–10^9^) was cyanidin 3‐*O*‐galactoside, which was increased by 3.17 folds from B to FM stage. The abundance of cyanidin *O*‐syringic acid was also 10^8^–10^9^, which showed a non significant increase during the maturation process. Contrast with this, the level of other types of cyanidins (cyanidin 3‐*O*‐glucoside, Cyanidin 3‐*O*‐(6‐O‐malonyl‐β‐d‐glucoside) and cyanidin 3,5‐diglucoside) were significantly decreased. Interestingly, the level of pelargonidin and pelargonidin 3‐*O*‐β‐d‐glucoside increased by 33.41 and 13.64 folds, respectively, but the abundance of all of the 4 peonidins (peonidin 3‐*O*‐glucoside chloride, peonidin *O*‐hexoside (glucoside), peonidin and peonidin 3,5‐diglucoside chloride) decreased. The above results indicated that cyanidin‐3‐*O*‐galactoside and other cyanidins were the predominant pigments in mature *Berchemia* fruit and increased accumulation of cyanidins and pelargonidins but decreased level of other anthocyanins might be the metabolic basis for purple pigmentation of *Berchemia* fruit.

**TABLE 2:** The anthocyanins and proanthocyanidins identified in *Berchemia* fruit pulp

Compounds	KEGG id	Abundance (peak area)		Log_2_FC	*p*‐value	Trend
		B	FM			
Cyanidin 3‐*O*‐galactoside Cyanidin *O*‐syringic acid Delphinidin 3‐*O*‐glucoside (Mirtillin) Pelargonidin Peonidin 3‐*O*‐glucoside (chloride) Peonidin *O*‐hexoside (glucoside) Pelargonidin 3‐*O*‐β‐d‐glucoside Petunidin 3‐*O*‐glucoside Cyanidin 3‐*O*‐glucoside (Kuromanin) Pelargonidin 3,5‐di‐β‐d‐glucoside Petunidin 3,5‐diglucoside Rosinidin *O*‐hexoside Peonidin Peonidin 3,5‐diglucoside (chloride) Cyanidin 3‐*O*‐(6‐*O*‐malonyl‐β‐d‐glucoside) Cyanidin 3,5‐*O*‐diglucoside (Cyanin)	C08647 – C12138 C05904 – C12141 C12137 C12139 C08604 C08725 – – C08726 – C12643 C08639	39,800,000 87,200,000 2,576,670 925,000 24,266,667 24,000,000 921,000 7,710,000 18,433,333 9 9 3,233,333 1,633,333 1,593,333 2,710,000 36,433,333	126,000,000 100,566,667 48,300,000 30,900,000 15,533,333 14,633,333 12,566,667 10,800,000 9,556,667 5,683,333 5,126,667 1,636,667 1,523,333 1,360,000 398,000 9	1.66 0.21 4.23 5.06 −0.64 −0.71 3.77 0.49 −0.95 19.27 19.12 −0.98 −0.10 −0.23 −2.77 −21.95	6.62E−05 8.36E−02 1.14E−05 8.87E−05 6.52E−03 7.87E−04 7.21E−05 6.63E−02 2.55E−02 7.52E−04 2.69E−05 8.16E−03 5.75E−01 3.62E−01 1.04E−07 5.11E−07	up NS up up NS NS up NS NS up up NS NS NS down down
Procyanidin A2 Procyanidin B2 Procyanidin B3	C10237 C17639 –	3,216,667 11,403,333 4,096,667	3,923,333 1,157,333 347,000	0.29 −3.30 −3.56	1.44E−01 2.38E−03 1.07E−03	NS down down

Note

up, up‐regulated; NS, not significant; down, down‐regulated.

### 3.6 The content of important medicinal components of *Berchemia* reported in previous references

The largest class of DAMs was flavonoid including 7 flavanones, 24 flavones, 9 flavonoid, 10 flavonols, and 1 isoflavone. In previous works, the biggest group of medicinal metabolites reported in *Berchemia* plant were flavonoid containing 1 flavone, 8 flavonols, 2 flavanones and 2 flavonoid (Bekker et al., 1996; Kikuchi et al., 1990; Lee et al., 1995; Shen, Teng, Yang, et al., 2010; Wang et al., 2006; Yang, Duan, et al., 2006). Among them, the accumulation of 4, 2′, 4′, 6′‐Tetrahydroxychalcone, kaempferol, dihydroquercetin, aromadendrin, naringenin, eriodictyol and 5, 7‐Dihydroxychromone in *Berchemia* fruits were up‐regulated from break stage to full‐mature stage (Table 3). Contrast with this, the content of glycosylated flavonoid such as quercetin 3‐*O*‐rutinoside (rutin), quercetin 3‐α‐arabinofuranoside, and kaempferol 3‐*O*‐glucoside were declined from break stage to full‐mature stage. Moreover, 6 polyphenols, 3 phenylpropanoids, 3 quinones, 2 organic acids and derivatives, 1 steride, and sucrose, which were identified in *Berchemia* leaves, wood, stem or root, were also detected in *Berchemia* fruit pulp. The above results indicated that the metabolic changes of *Berchemia* fruit during maturation might result in changes of pharmaceutical effect which need a further investigation.

**TABLE 3:** The contents of metabolites from *Berchemia floribunda* fruits reported in previous documents

Class	Compounds	References^a^	MW/Da	KEGG id	Log_2_FC	*p*‐value
Polyphenol	Gallocatechin Epigallocatechin (EGC) l‐Epicatechin Protocatechuic acid *O*‐glucoside Catechin Protocatechuic acid	7 14 6 4 4, 6, 7, 14 4	306.07 306.0 290.3 316.1 290.08 154.03	C12127 C12136 C09727 – C06562 C00230	−5.33 −2.79 −2.75 −2.44 −2.36 −0.58	2.57E−04 7.53E−04 6.10E−04 1.55E−03 1.93E−03 1.41E−02
Flavone Flavonol	4,2′,4′,6′‐Tetrahydroxychalcone	8	272.07	C06561	4.55	7.72E−03
	Quercetin Quercetin 3‐*O*‐rutinoside (Rutin) Quercetin 3‐α‐arabinofuranoside Kaempferol 3‐*O*‐glucoside Myricetin 3‐*O*‐rhamnoside Kaempferol Dihydroquercetin (Taxifolin) Aromadendrin	4, 7, 10, 12, 14 4, 7, 10 12 4 4 4, 12 4, 7, 12, 14 7, 12, 14	302.04 610.15 434.08 448.1 464.1 286.05 304.06 288.06	C00389 C05625 – C12249 C10108 C05903 C01617 C00974	−0.46 −0.40 −0.21 −0.21 0.50 1.03 5.12 6.13	1.05E−02 5.76E−03 3.93E−01 3.28E−02 8.18E−04 1.68E−01 1.18E−05 1.04E−05
Flavanone	Naringenin Eriodictyol	4, 7, 8,14 4, 12, 14	272.07 288.06	C00509 C05631	5.92 20.41	6.65E−04 4.66E−04
Flavonoid	Narcissoside (narcissin) 5, 7‐Dihydroxychromone	4 10	624.17 178.03	– C09001	−0.66 3.79	3.94E−03 7.94E−03
Phenylpropanoid	Ferulic acid Vanillic acid Phillygenin (Phillyroside)	13 4 10	194.06 168.0 534.21	C01494 C06672 C17048	−0.45 −2.18 −2.38	1.65E−01 4.23E−04 5.80E−03
Quinones	Aurantio‐obtusi Chrysophanol Emodin	13 11, 15 10	330.29 254.06 270.05	C17670 C10315 C10343	−0.93 0.53 3.13	9.87E−03 5.80E−02 6.84E−02
Organic acids & derivatives	Syringic acid *O*‐glucoside 4‐Hydroxybenzoic acid	3 4	360.10 138.03	– C00156	−1.48 −1.31	1.13E−04 1.40E−05
Sterides	β‐Sitosterol	4, 11	414.39	C01753	−0.48	1.45E−01
Carbohydrates	d‐(+)‐Sucrose	4	342.12	C00089	1.99	1.05E−04

^a^ Reference 3 (Inoshiri et al., 1988), 5 (Inoue et al., 1990) & 6 (Sakurai et al., 1992): *B. racemosa* SIEB. *et* ZUCC, stem; Reference 4 (Kikuchi et al., 1990): *B. racemosa* SIEB. *et* ZUCC, leaves & wood; Reference 7 (Lee et al., 1995): *B. formosana*: Stem & root; Reference 8 (Bekker et al., 1996), 14 (Shen, Teng, Yang, et al., 2010) & 15 (Shen et al., 2010): *B. lineata* (L.) DC.: root; Reference 10 (Yang, Pan, et al., 2006), 11 (Yang, Duan, et al., 2006) & 13 (Jing et al., 2011): *B. polyphylla* var. *leioclada*, whole plant; Reference 12 (Wang et al., 2006): barks of *B. floribunda* (Wall.) Brongn.

## 4 CONCLUSION

Although dozens of metabolites have been reported in leaves, stems, root, wood, bark and whole plant of *Berchemia* (Rhamnaceae) species, no comprehensive evaluation of the metabolites in *Berchemia* floribunda fruit has been reported. In the present study, a large‐scaled detection of metabolites in *Berchemia floribunda* fruit pulp were conducted by a widely targeted metabolic analysis. 730 metabolites were detected and 644 metabolites were identified in *B. floribunda* fruit pulp. The highest total flavonoid and total phenolic content in *B. floribunda* fruit pulp were found at red ripe stage but the total anthocyanin content was highest at full‐mature stage. During the ripening process, 123 metabolites were up‐regulated and 111 metabolites were down‐regulated, and the DAMs were enriched into biosynthesis of phenylpropanoids, flavonoid, and alkaloids. The unripe *B. floribunda* fruit showed relative high contents of the previous reported pharmaceutical compounds when compared to those in full‐mature fruits. More importantly, increased accumulation of cyanidins and pelargonidins but decreased level of other anthocyanins might be the metabolic basis for purple pigmentation of full‐mature *Berchemia* fruit. Thus, the unripe fruit should be used in medicine, while the full‐mature fruits were suggested to be used in food products. In summary, this study outlines the first comprehensive metabolic fingerprint of *B. floribunda* fruits which might benefit the further study of medicinal components and edible pigments of *B. floribunda* fruits.

## CONFLICT OF INTEREST

The authors declare that they have no conflicts of interest.

## ETHICAL APPROVAL

This study does not involve any human or animal testing.
